# Nonsurgical Management of Cardiac Papillary Fibroelastoma on the Aortic Valve

**DOI:** 10.14740/jmc5247

**Published:** 2026-01-13

**Authors:** Yonis Hakim, Yousef Raslan Hakim, William Barker, Tariq Ahmad

**Affiliations:** a Medical Education, Tulane University School of Medicine, New Orleans, LA, USA; b Internal Medicine Department, St. Luke’s Hospital – Anderson Campus, Easton, PA, USA; c Family Medicine Department, Allina Health United Family Physicians Clinic, St. Paul, MN, USA; d Cardiology Department, Interventional Cardiology, Geisinger Wyoming Valley Medical Center, Wilkes-Barre, PA, USA

**Keywords:** Papillary fibroelastoma, Cardiac neoplasm, Aortic valve, Nonsurgical management, Long-term anticoagulation, Apixaban, Myocardial infarction, ST-elevation myocardial infarction

## Abstract

Papillary fibroelastomas are benign cardiac tumors that constitute the second most common cardiac tumors. Controversy exists in the management of papillary fibroelastoma. When to surgically manage the patient or use pharmaceutical therapy is not clear. There are studies that indicate that nonsurgical management might be associated with higher mortality and morbidity rates and more adverse events. There has not been a reported case of papillary fibroelastoma managed successfully with only anticoagulation. Clearer guidelines are needed for the management of papillary fibroelastoma, especially in cases where a patient is a poor surgical candidate or declines surgical intervention. In this case, a patient has been managed nonsurgically for 4 years and 7 months up to date. The patient is a 57-year-old female who presented to the emergency department with myocardial infarction symptoms. The myocardial infarction was thought to be secondary to an embolic event after a patent foramen ovale was identified on transthoracic echocardiogram or sequelae from arrhythmia. Cardionet ruled out arrhythmia, and patent foramen ovale closure workup revealed a 0.3-cm mobile papillary fibroelastoma. Surgical management was not pursued due to surgical risks and the patient’s preference, and the patient was prescribed long-term apixaban. The patient was followed for 4 years and 7 months and experienced an episode of vaginal bleeding during this time. This case shows an example of when nonsurgical management can be pursued as the patient declined surgical intervention after benefits and risks were discussed. Also, this case shows the importance of considering the patient’s bleeding risk, such as this patient’s history of hematuria due to acute cystitis, miscarriages, and heparin-induced gingival hematoma while hospitalized, prior to initiating anticoagulation. Bleeding risk can be assessed using the HAS-BLED risk score or equivalent.

## Introduction

Papillary fibroelastomas are benign cardiac tumors that constitute the second most common cardiac tumors [1]. Recent data, however, indicate that they may be more frequent than myxomas, the most common cardiac tumors [2]. Papillary fibroelastomas can be asymptomatic or present as transient ischemic attack (TIA) or ischemic stroke, and they can have cardiovascular manifestations, including angina and myocardial infarction (MI) [1]. They are diagnosed via echocardiography and affect the aortic valve most commonly [1]. Currently, the medical literature does not provide clear guidelines for the management of papillary fibroelastoma. Guidelines are not clear regarding when to surgically manage the patient or use conservative management [3]. In this case, we report an instance of a patient who was nonsurgically managed because the patient declined surgical intervention.

## Case Report

### Investigations

A 57-year-old female patient presented to the emergency department (ED) with classical MI symptoms. The patient presented with acute sharp, stabbing, left-sided chest pain, as well as left shoulder and upper back pain radiating to the left arm. The pain started during exercise and lasted for 1 h. It was associated with near-syncope, nausea, and diaphoresis. The patient did not experience dyspnea. The past medical history of the patient was notable for chronic hypertension, cervical radiculopathy, laryngopharyngeal reflux, miscarriages, and acute cystitis with hematuria. The patient had a family history notable for abdominal aortic aneurysm.

### Diagnosis

The differential diagnosis for this patient included MI, unstable angina, stable angina, pulmonary embolus, aortic dissection, esophagitis, and peptic ulcer disease. The medical team started to evaluate the chest pain. An electrocardiogram (EKG) study was performed for the patient. This EKG showed inferior ST-elevation myocardial infarction (STEMI). Nitroglycerin was not administered due to concerns of ventricular involvement. The patient was given aspirin, ticagrelor, heparin, and atorvastatin in the ED. The patient also had a transthoracic echocardiogram (TTE) and coronary angiogram performed. Coronary angiogram showed flush occlusion in the extremely distal apical left anterior descending (LAD) artery, which gave off the left posterior descending artery (PDA). TTE also showed small right-to-left shunting via a patent foramen ovale (PFO), which was incidentally found. Given the findings, embolic coronary phenomenon was suspected. Due to the occlusion’s being more distal than possible for intervention, interventional cardiology and hematology recommended hypercoagulability workup and long-term anticoagulation. The patient was administered aspirin, heparin, nitroglycerin, verapamil, and high-intensity statin and was admitted for 3 days.

A duplex study of the bilateral lower extremities to rule out a paradoxical embolus was performed. This study, a chest X-ray, and blood work, including hypercoagulable workup, were predominantly negative. While the patient was hospitalized, a gingival hematoma developed, so unfractionated heparin was held.

### Treatment

**Figure 1:**
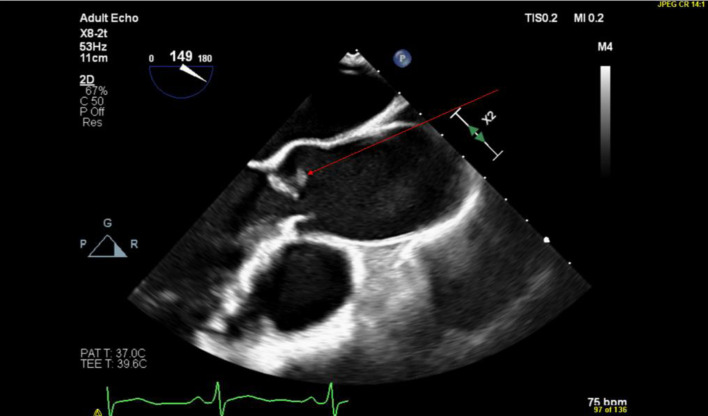
TEE showing a parasternal long axis view of the aortic valve with the papillary fibroelastoma indicated by the red arrow. TEE: transesophageal echocardiogram.

The medical team performed a holistic review of the patient’s past medical history, hospital course, and diagnostic studies. Then, the medical team discussed available treatment options with the patient to reach a decision regarding the patient’s treatment plan. With the patient’s consent, the medical team made the decision to manage the patient with long-term anticoagulation due to the difficulty of intervention on the distal apical LAD artery. The patient was discharged on apixaban. The Cardiology team followed up with the patient. A Cardionet study was performed to rule out arrythmias as a cause of the embolic phenomenon with the PFO as a conduit. The Cardionet study was mostly unremarkable, with the exception of sinus bradycardia related to beta blockers. The Cardiology team proceeded with PFO closure assessment. For better illustration of the PFO, which was demonstrated on the transthoracic echocardiography that was performed while the patient was hospitalized, the cardiology team ordered a transesophageal echocardiogram (TEE). The TEE was performed 2 months after discharge and showed a small 0.3 cm mobile echodensity on the right coronary aortic valve leaflet with stalk attachment and areas of echolucency within the tumor. These echocardiographic features were suggestive of an incidental finding of a papillary fibroelastoma on the aortic valve (Fig. 1). The TEE also demonstrated the previous finding of right to left shunting through the PFO, which was previously demonstrated on TTE.

Due to the papillary fibroelastoma, cardiology did not proceed with the closure of the patient’s PFO. Benefits and risks of surgical intervention were discussed with the patient. Surgical risks, such as valvular damage during surgery and lesion embolization, were explained to the patient. The benefits of avoiding morbidity and lowering the risks of stroke and MI were also discussed. The patient preferred to avoid surgical intervention. Given the patient’s decision, management with long-term anticoagulation with apixaban was chosen. The patient also tolerated apixaban for 2 months prior to diagnosing the papillary fibroelastoma, which supported the decision to nonsurgically manage the patient with close follow-up. The management plan was formed with the involvement of cardiology, interventional cardiology, and primary care. The patient also sought second opinion at a tertiary cardiovascular center that is a leading center in the region. The patient was recommended to continue apixaban therapy.

### Follow-up and outcomes

Two months after discharge from the hospital, the patient experienced an episode of heavy, non-life-threatening vaginal bleeding, with associated pelvic pain and cramping. The patient saturated one pad per hour. The patient was hemodynamically stable and did not require hospitalization. The bleeding was due to leiomyomata and adenomyosis. The patient was prescribed medroxyprogesterone and followed up with the obstetrics/gynecology for intrauterine device (IUD) placement.

The patient also experienced three episodes of neck and shoulder pain during the prolonged period of follow-up of 4 years and 7months due to cervical radiculopathy. The pain was reproducible on physical exam, and MI was excluded with EKG.

In the most recent follow-up, 4 years after the patient’s initial presentation, the patient expressed that she would like to consider stopping anticoagulation and pursuing an alternative treatment plan. If the patient decides to undergo surgical interventions, surgical resection of the papillary fibroelastoma will be considered if it is still present on repeat TEE and upon closure of the PFO.

## Discussion

Current guidelines for the management of papillary fibroelastoma are not clear. There are various recommendations posited by researchers, and when to surgically or nonsurgically manage the patient is not clear [3]. Of note, however, there has not been a reported case of papillary fibroelastoma managed successfully with only anticoagulation [4]. This case described herein shows an example of a papillary fibroelastoma that was managed nonsurgically with anticoagulation. In our case, the patient was specifically managed with apixaban anticoagulation. This case clarifies a situation when nonsurgical management can be pursued successfully.

In our case, the patient would have qualified for surgical management per some recommendations as the patient was symptomatic, a good surgical candidate, and had a mobile lesion. In the case of left-sided papillary fibroelastomas, which is the case of our patient, surgical resection is recommended if the patient is symptomatic [4–6]. There are other recommendations to surgically resect left-sided lesions if the patient is solely a good surgical candidate [7]. Also, there are recommendations to resect if the lesion is highly mobile or large, given increased cardiovascular complications and cardiac death with large and/or mobile lesions [6, 8]. Given the aforementioned recommendations, the patient would have qualified for surgery.

Surgical interventions, nonetheless, carry risks, such as valvular damage and fragmentation of the lesion [1, 9, 10]. The likelihood of valvular injury is significant, particularly with excision of larger lesions [10]. Among 325 patients who underwent surgical resection of papillary fibroelastoma, 39 patients required valve repair, and 42 patients required valve replacement [1].

Surgical interventions, however, have the advantages of low recurrence rates, in addition to the lower risks of mortality and adverse effects. In a study on 325 papillary fibroelastoma patients who were treated surgically, there was no recurrence [1]. In another study of 21 patients with papillary fibroelastoma managed surgically, there was no recurrence with an average follow-up of 17 ± 14 months [11]. Regarding mortality and adverse effects, in another study, patients who were managed surgically had a lower risk of cerebrovascular accidents than patients who did not undergo surgery [7]. In those managed surgically, the cerebrovascular risk at 1- and 5-year follow-up was 2% and 8%, respectively [7]. On the other hand, in those who did not undergo surgery, the cerebrovascular risk at 1- and 5-year follow-up was 6% and 13%, respectively [7]. There was also a difference in survival rates between patients who were treated surgically, in contrast to those patients who did not undergo surgery [7]. The survival rates at 1- and 5-year follow-up for the patients who were managed surgically were 98% and 84%, respectively [7]. On the other hand, survival rates for the patients who did not undergo surgery at 1- and 5-year follow-up were 87% and 67%, respectively [7]. There is a study, however, that found that long-term survival between surgically and nonsurgically managed patients was similar [12]. Given the above, surgical interventions have lower risk of mortality and adverse events.

After discussing the benefits and risks with the patient, the patient preferred to avoid surgery, given risks such as valvular damage. Given that the patient declined surgical intervention, there are recommendations to start pharmacologic management [7, 13]. These recommendations are given by researchers in the cases of the patients’ declining surgical management or being poor surgical candidates [7, 13]. Nonetheless, nonsurgical management carries the risks of increased mortality, morbidity, and adverse events, as mentioned above.

In this case, the team concluded that long-term anticoagulation may be well tolerated with close follow-up. This was based on the fact that the patient was managed successfully over a 2-month period on anticoagulation by the time of finding the papillary fibroelastoma on imaging. The patient also sought a second opinion at a tertiary center, which agreed with the management plan.

This case, nonetheless, is not intended to promote nonsurgical management for all patients with left-sided papillary fibroelastoma. As mentioned above, nonsurgical management carries greater risks. This case is intended to clarify when nonsurgical management can be safely pursued. In this case, the patient was young with few comorbidities and had tolerated anticoagulation prior to finding the papillary fibroelastoma. The patient also declined surgical intervention. In such case or when the patient is a poor surgical option, nonsurgical treatment will need to be pursued. More research is needed to clarify when nonsurgical management can be pursued safely.

It is important to note that the patient experienced an episode of vaginal bleeding around two and a half months after initiation of anticoagulation. To avoid this, bleeding risk can be assessed at the initiation of anticoagulation and routinely upon follow-up using the HAS-BLED risk score or similar assessment tools in high-risk patients [14]. The HAS-BLED risk score is briefer than the HEMOR_2_RHAGES risk score and demonstrates similar accuracy [15]. After initiation of anticoagulation, routine anticoagulation monitoring is not required and may not be advantageous [14]. However, it may be prudent to consider it, especially if the patient has a bleeding history or bleeding susceptibility as with the patient herein, who had a history of miscarriages and heparin-induced gingival hematoma.

In conclusion, papillary fibroelastomas are common cardiac tumors. Management guidelines are variable. This report illustrates the need for more research on the management guideline, especially for therapeutic nonsurgical management. This case report also offers a situation when nonsurgical management can be successfully pursued. The patient described herein has been successfully managed for 4 years and 7 months to date.

### Learning points

Clearer guidelines are needed for the management of papillary fibroelastoma, especially for nonsurgical management. In the case of nonsurgical management, it is also important to consider the patient’s bleeding risk, prior to initiating anticoagulation. The bleeding risk can be assessed using the HAS-BLED risk score or similar assessment methods.

## Data Availability

Data sharing is not applicable to this article.

## Abbreviations

EKG: electrocardiogram
ED: emergency department
IUD: intrauterine device
LAD: left anterior descending
MI: myocardial infarction
PFO: patent foramen oval
PDA: posterior descending artery
STEMI: ST-elevation myocardial infarction
TEE: transesophageal echocardiogram
TIA: transient ischemic attack
TTE: transthoracic echocardiogram
